# Supplementary material for: Innexin function dictates the spatial relationship between distal somatic cells in the Caenorhabditis elegans gonad without impacting the germline stem cell pool
Source: eLife. 2022 Sep 13;11:e74955. doi: 10.7554/eLife.74955 (PMC9473689; doi:10.7554/eLife.74955)
Supplement: Supplementary file 1. [file elife-74955-supp1.docx]

**Strain Genotype Reference**

**AG400** *fasn-1(av138[fasn-1::gfp]) I* (Starich et al. 2020)

**CB190** *unc-54(e190) I* (Brenner 1974)

**CB1282** *dpy-20(e1282) IV* (Hosono et al. 1982)

**DG2506** *acy-4(ok1806) V; tnEx42[acy-4::gfp + rol-6(su1006)]* (Govindan et al. 2009)

**DG4959** *qIs154[lag-2p::MYR::tdTomato + ttx-3p::gfp] V;*

*tnIs5[lim-7p::gfp + rol-6(su1006)] X* (this work)

**DG4977** *inx-14(ag17) I; qIs154[lag-2p::MYR::tdTomato + ttx-3p::gfp] V;*

*tnIs5[lim-7p::gfp + rol-6(su1006)] X* (this work)

**DG5020** *naIs37[lag-2p::mCherry::PH + unc-119(+)] I;*

*bcIs39[lim-7p::ced-1::gfp + lin-15(+)] V* (this work)

**DG5026** *inx-14(ag17) naIs37[lag-2p::mCherry::PH + unc-119(+)] I;*

*bcIs39[lim-7p::ced-1::gfp + lin-15(+)] V* (this work)

**DG5027** *naIs37[lag-2p::mCherry::PH + unc-119(+)] I;*

*inx-9(ok1502) IV; bcIs39[lim-7p::ced-1::gfp + lin-15(+)] V* (this work)

**DG5029** *naIs37[lag-2p::mCherry::PH + unc-119(+)] I;* *inx-8(tn1513tn1555) inx-9(ok1502) IV;*

*bcIs39[lim-7p::ced-1::gfp + lin-15(+)] V* (this work)

**DG5059** *inx-9(ok1502) IV* (this work)

**DG5063** *inx-8(qy78[mKate2::inx-8]) IV* (this work)

**DG5064** *inx-8(qy102(mKate2::inx-8)) inx-9(ok1502) IV* (this work)

**DG5070** *inx-14(ag17) I; inx-8(qy78[mKate2::inx-8]) IV* (this work)

**DG5131** *naIs37[lag-2p::mCherry::PH + unc-119(+)] I;inx-8(qy78[mKate2::inx-8]) IV;*

*bcIs39[lim-7p::ced-1::gfp + lin-15(+)] V* (this work)

**DG5133** *naIs37[lag-2p::mCherry::PH + unc-119(+)] I;* *inx-8(qy102(mKate2::inx-8))*

*inx-9(ok1502) IV; bcIs39[lim-7p::ced-1::gfp + lin-15(+)] V* (this work)

**DG5136** *sygl-1(q983[3xOLLAS::sygl-1]) naIs37[lag-2p::mCherry::PH + unc-119(+)] I;*

*bcIs39[lim-7p::ced-1::gfp + lin-15(+)] V* (this work)

**DG5150** *inx-14(ag17) sygl-1(q983[3xOLLAS::sygl-1]) naIs37[lag-2p::mCherry::PH + unc-119(+)] I; bcIs39[lim-7p::ced-1::gfp + lin-15(+)] V* (this work)

**DG5181** *sygl-1(q983[3xOLLAS::sygl-1]) naIs37[lag-2p::mCherry::PH + unc-119(+)] I;*

*inx-8(qy78[mKate2::inx-8]) IV;*

*bcIs39[lim-7p::ced-1::gfp + lin-15(+)] V* (this work)

**DG5229** *naIs37[lag-2p::mCherry::PH + unc-119(+)] I;* *inx-8(qy78tn2031) IV;*

*bcIs39[lim-7p::ced-1::gfp + lin-15(+)] V* (this work)

**DG5232** *naIs37[lag-2p::mCherry::PH + unc-119(+)] I;* *inx-8(tn2034) IV;*

*bcIs39[lim-7p::ced-1::gfp + lin-15(+)] V* (this work)

**DG5248** *sygl-1(q983[3xOLLAS::sygl-1]) naIs37[lag-2p::mCherry::PH + unc-119(+)] I;*

*inx-8(qy78tn2031) IV; bcIs39[lim-7p::ced-1::gfp + lin-15(+)] V* (this work)

**DG5249** *sygl-1(q983[3xOLLAS::sygl-1]) naIs37[lag-2p::mCherry::PH + unc-119(+)] I;*

*inx-8(tn2034) IV; bcIs39[lim-7p::ced-1::gfp + lin-15(+)] V* (this work)

**DG5250** *inx-8(qy78tn2031) IV* (this work)

**DG5251** *inx-8(tn2034) IV* (this work)

**DG5270** *inx-14(ag17) I* (this work)

**DG5310** *naIs37[lag-2p::mCherry::PH + unc-119(+)] I; acy-4(ok1806) V;*

*tnEx42[acy-4::gfp + rol-6(su1006)]* (this work)

**DG5320** *fasn-1(av138[fasn-1::gfp])*

*naIs37[lag-2p::mCherry::PH + unc-119(+)] I* (this work)

**DG5346** *naIs37[lag-2p::mCherry::PH + unc-119(+)] I;*

*inx-8(qy78[mKate2::inx-8])/tmC5[tmIs1220] IV;*

*bcIs39[lim-7p::ced-1::gfp + lin-15(+)] V* (this work)

**DG5347** *naIs37[lag-2p::mCherry::PH + unc-119(+)] I;*

*inx-8(qy78tn2031)/tmC5[tmIs1220] IV;*

*bcIs39[lim-7p::ced-1::gfp + lin-15(+)] V* (this work)

**DG5357** *tmC5[tmIs1220] inx-8(tn2075) IV* (this work)

**DG5366** *naIs37[lag-2p::mCherry::PH + unc-119(+)] I;*

*inx-8(qy78[mKate2::inx-8])/tmC5[tmIs1220] inx-8(tn2075) IV;*

*bcIs39[lim-7p::ced-1::gfp + lin-15(+)] V* (this work)

**DG5367** *inx-14(ag17) fasn-1(av138[fasn-1::gfp])*

*naIs37[lag-2p::mCherry::PH + unc-119(+)] I* (this work)

**DG5378** *fasn-1(av138[fasn-1::gfp]) naIs37[lag-2p::mCherry::PH + unc-119(+)] I;*

*inx-8(qy78[mKate2::inx-8]) IV* (this work)

**DG5380** *bcIs39[lim-7p::ced-1::gfp + lin-15(+)] V* (this work)

**FX30140** *tmC5[tmIs1220] IV* (Dejima et al. 2018)

**JK1466** *gld-1(q485)/dpy-5(e61) unc-32(e51) I* (Francis et al. 1995)

**KLG006** *inx-8(qy78[mKate2::inx-8]) IV; tnIs6[plim-7::gfp + rol-6(su1006)] X;*

*cpIs122(lag-2p::mNeonGreen::plcdeltaPH)* (Gordon et al., 2020)

**NK2571** *inx-8(qy78[mKate2::inx-8]);*

*cpIs122 [lag-2p::mNeonGreen::plcdeltaPH]* (Gordon et al., 2020)

**NK2576** *inx-8(qy102(mKate2::inx-8)) inx-9(ok1502);*

*cpIs122(lag-2p::mNeonGreen::plcdeltaPH)* (Gordon et al., 2020)
