# Supplementary material for: Innexin function dictates the spatial relationship between distal somatic cells in the Caenorhabditis elegans gonad without impacting the germline stem cell pool
Source: eLife. 2022 Sep 13;11:e74955. doi: 10.7554/eLife.74955 (PMC9473689; doi:10.7554/eLife.74955)
Supplement: Supplementary file 2. [file elife-74955-supp2.docx]

**Table S 2. Oligonucleotides used in this study**

**Oligo name Sequence**

AF-ZF-827 CACTTGAACTTCAATACGGCAAGATGAGAATGACTGGAAACCGT

ACCGCATGCGGTGCCTATGGTAGCGGAGCTTCACATGGCTTCAG

ACCAACAGCCTAT (Arribere et al., 2014)

inx8_us_sgRNA1.F TCTTGTGGAAAACAGAGGAATGGG

inx8_us_sgRNA1.R AAACCCCATTCCTCTGTTTTCCAC

inx8_sgRNA1.F TCTTGAGTGACTTGGTAGCATCGG

inx8_sgRNA1.R AAACCCGATGCTACCAAGTCACTC

inx8_RPR GGTGGCCAATAAAAATGCTTTTCTTTTTGCTTTT

CTCTATCTACTTCCGTTCCGCCCCGGAGGTTGCC

GTGGAGATGTACAGCGACTTTTTAGTAAGTCTTT

TCAAC

inx8_delta.F CCTTCGACCTGATTTCCCCTTCTTCTAATG

inx8_delta.R CTATTGCTTTCCGTTCTTCAAGATGTTGTTG

inx-14delF GATACGACGTGAGCAATGGAACGTC

inx-14delR CTTGGACTTGAAGTGAGAGTTGGAG

sygl1-F ATCATCGAACCATTGTCATCACGC

sygl1-R TTTGCCTTGATCTCCAAGTGTTGC
