# Supplementary material for: Innexin function dictates the spatial relationship between distal somatic cells in the Caenorhabditis elegans gonad without impacting the germline stem cell pool
Source: eLife. 2022 Sep 13;11:e74955. doi: 10.7554/eLife.74955 (PMC9473689; doi:10.7554/eLife.74955)
Supplement: Transparent reporting form [file elife-74955-transrepform1.docx]

***eLife’s* transparent reporting form**

We encourage authors to provide detailed information *within their submission* to facilitate the interpretation and replication of experiments. Authors can upload supporting documentation to indicate the use of appropriate reporting guidelines for health-related research (see [EQUATOR Network](http://www.equator-network.org/%20)), life science research (see the [BioSharing Information Resource](https://biosharing.org/)), or the [ARRIVE guidelines](http://www.plosbiology.org/article/info:doi/10.1371/journal.pbio.1000412) for reporting work involving animal research. Where applicable, authors should refer to any relevant reporting standards documents in this form.

If you have any questions, please consult our Journal Policies and/or contact us: [editorial@elifesciences.org](mailto:editorial@elifesciences.org).

**Sample-size estimation**

- You should state whether an appropriate sample size was computed when the study was being designed
- You should state the statistical method of sample size computation and any required assumptions
- If no explicit power analysis was used, you should describe how you decided what sample (replicate) size (number) to use

Please outline where this information can be found within the submission (e.g., sections or figure legends), or explain why this information doesn’t apply to your submission:

For Table 1, sample size is indicated and additional information is provided in Materials and Methods.

Figures 1 and 2 and related supplements are live samples, with the exception of Fig. 2B, which contains fixed samples. Typical sample sizes for live samples are 10-27 individuals (Byrd et al. 2014 https://dx.plos.org/10.1371/journal.pone.0088372; Linden et al. 2017 http://linkinghub.elsevier.com/retrieve/pii/S0012160617301902). We have conformed to this convention for Figs. 1 and 2. Sample sizes are stated in Figures, legends, and/or in text, where applicable.

Figure 2B and Figures 3 and 4 and related supplements are fixed samples. For the assessment of protein expression in fixed samples, at least 15 gonads in 1-4 biological replicates are typically examined in the field (Seidl & Kimble 2015 [https://doi.org/10.7554/eLife.10832.001](https://urldefense.com/v3/__https:/doi.org/10.7554/eLife.10832.001__;!!MXfaZl3l!JKtYKxaHfUzoEBan4olm2n71xwuuD0IRUxG08Lnso-hSVKuv0CGiIL9qRWmo76AOPn5LWcMC$); Singh et al., 2021 [https://doi.org/10.1371/journal.pgen.1009687](https://urldefense.com/v3/__https:/doi.org/10.1371/journal.pgen.1009687__;!!MXfaZl3l!JKtYKxaHfUzoEBan4olm2n71xwuuD0IRUxG08Lnso-hSVKuv0CGiIL9qRWmo76AOPpoe0Rpq$)). We have conformed to this convention for Figs. 3 and 4 and their supplements. Sample sizes are stated in Figures, legends, and/or in text, where applicable.

**Replicates**

- You should report how often each experiment was performed
- You should include a definition of biological versus technical replication
- The data obtained should be provided and sufficient information should be provided to indicate the number of independent biological and/or technical replicates
- If you encountered any outliers, you should describe how these were handled
- Criteria for exclusion/inclusion of data should be clearly stated
- High-throughput sequence data should be uploaded before submission, with a private link for reviewers provided (these are available from both GEO and ArrayExpress)

Please outline where this information can be found within the submission (e.g., sections or figure legends), or explain why this information doesn’t apply to your submission:

For the brood count assays, the number of independent assessments is indicated by the “n values” in Table 1. The source data for Table 1 is provided in the accompanying spreadsheet.

For Figure 1 and 2 and their supplements, we define a biological replicate as a set of individuals collected and imaged in the same session. Numbers of individuals included in each analysis is indicated in the figure legends.

For Figure 3 and 4, and their supplements, we define a biological replicate as independently dissected and stained, along with the controls in the same tube, and images acquired with the same settings. During these experiments, no outliers encountered. In rare cases, acquired images, which either failed to stitch by Fiji or had poor staining, were discarded.

**Statistical reporting**

- Statistical analysis methods should be described and justified
- Raw data should be presented in figures whenever informative to do so (typically when N per group is less than 10)
- For each experiment, you should identify the statistical tests used, exact values of N, definitions of center, methods of multiple test correction, and dispersion and precision measures (e.g., mean, median, SD, SEM, confidence intervals; and, for the major substantive results, a measure of effect size (e.g., Pearson's r, Cohen's d)
- Report exact p-values wherever possible alongside the summary statistics and 95% confidence intervals. These should be reported for all key questions and not only when the p-value is less than 0.05.

Please outline where this information can be found within the submission (e.g., sections or figure legends), or explain why this information doesn’t apply to your submission:

The statistical tests used in Figures 1–4 and the N and p values are reported in the respective figure legends and/or in the figures themselves.

(For large datasets, or papers with a very large number of statistical tests, you may upload a single table file with tests, Ns, etc., with reference to sections in the manuscript.)

**Group allocation**

- Indicate how samples were allocated into experimental groups (in the case of clinical studies, please specify allocation to treatment method); if randomization was used, please also state if restricted randomization was applied
- Indicate if masking was used during group allocation, data collection and/or data analysis

Please outline where this information can be found within the submission (e.g., sections or figure legends), or explain why this information doesn’t apply to your submission:

This information is provided in the Materials and Methods section.

**Additional data files (“source data”)**

- We encourage you to upload relevant additional data files, such as numerical data that are represented as a graph in a figure, or as a summary table
- Where provided, these should be in the most useful format, and they can be uploaded as “Source data” files linked to a main figure or table
- Include model definition files including the full list of parameters used
- Include code used for data analysis (e.g., R, MatLab)
- Avoid stating that data files are “available upon request”

Please indicate the figures or tables for which source data files have been provided:

All data is presented in the figures, the tables, and the accompanying source data.

We have provided source data for Table 1, Figure 1, Figure 1—supplement 1, Figure 2, Figure 2—supplement 2, Figure 2—supplement 3, Figure 3 and Figure 3—supplement 1 (all data for main and supplemental figure are in a single data file), Figure 4 and Figure 4—supplement 1 (all data for main and supplemental figure are in a single data file).
